# Balance Abilities and Kinesiophobia in Women with Fibromyalgia Syndrome: A Cross-Sectional Comparative Study

**DOI:** 10.5152/ArchRheumatol.2025.11155

**Published:** 2025-09-01

**Authors:** Cem Zafer Yıldır, Ejder Berk, Vedat Nacitarhan, Burhan Fatih Koçyiğit, Tuba Tülay Koca, Adnan Demirel

**Affiliations:** 1 Department of Physical Medicine and Rehabilitation, Kahramanmaraş Sütçü İmam University Training and Research Hospital, Kahramanmaraş, Türkiye; 2 Department of Physical Medicine and Rehabilitation, İzzet Baysal Physical Treatment and Rehabilitation Training and Research Hospital, Bolu, Türkiye

**Keywords:** Fear of movement, fibromyalgia, kinesiophobia, postural balance, women

## Abstract

**Background/Aims:**

This study evaluated balance performance and kinesiophobia levels between fibromyalgia syndrome patients and healthy controls to establish their relationship.

**Materials and Methods:**

Sixty female patients diagnosed with fibromyalgia and 60 healthy volunteers who did not have the condition were included in the study. The Fibromyalgia Impact Questionnaire was applied to the participants to evaluate the disease activity, and the Tampa Kinesophobia Scale was used for the evaluation of kinesiophobia. The four-square stepping test (FSST), functional reach test, timed up and go test (TUG), and posturography device were used to evaluate balance. The Mann–Whitney *U* test was used for comparing continuous variables between groups, the chi-square test for categorical variables, and Spearman’s rank correlation for examining relationships between parameters, with significance set at *P* < .05.

**Results:**

The fibromyalgia syndrome patients demonstrated significantly impaired balance abilities and elevated kinesiophobia scores compared to control subjects (*P* < .001). The FMS group experienced significantly more falls during the 6-month period than the control group, which had no falls (*P* < .001). Fall distribution showed that 30 patients (50%) experienced falls, with 18 patients having 1 fall, 8 patients having 2 falls, and 4 patients having ≥3 falls. Of the 60 FMS patients, 50 (83.3%) used medications with various combinations. The Tampa Scale for Kinesiophobia scores showed a statistically significant relationship with Fibromyalgia Impact Questionnaire scores (r: 0.507, *P* < .001). The balance parameters and kinesiophobia scores of FMS patients were both impaired, yet no significant relationship existed between these 2 measures (*r* values ranging from 0.08 to 0.15, all *P* > .05). Clinical balance tests (TUG, FSST) and most posturographic parameters failed to show any statistical connection with FMS disease activity.

**Conclusion:**

The results showed that female FMS patients had significantly impaired balance and higher kinesiophobia scores than healthy controls. These results show that balance impairments and kinesiophobia are both present in FMS, but they seem to be different aspects of the condition rather than directly related. Both factors should be assessed independently in clinical evaluation. Future research should investigate the mechanisms of these separate but co-occurring impairments in FMS.

**Main Points**

- FMS patients demonstrated significantly impaired static and dynamic balance compared to healthy controls.
- Kinesiophobia levels were markedly higher in FMS patients and showed a moderate correlation with disease activity (FIQ scores). Fifty percent of FMS patients experienced at least one fall over six months, whereas controls reported no falls.
- There was no significant association between kinesiophobia and objective balance measures, suggesting distinct underlying mechanisms.
- Clinical assessment of FMS should include independent evaluation of both balance impairments and movement-related fear.

## Introduction

Fibromyalgia syndrome (FMS) is a chronic condition characterized by widespread pain, disrupted sleep, fatigue, cognitive difficulties, and mood disturbances. This disorder significantly impairs patients' quality of life, necessitating continued research and improving knowledge about this condition. Although the prevalence of FMS varies between 2% and 8% according to the selected diagnostic criteria, it is generally accepted as 2%.^1,2^ Although fibromyalgia is more common in women between the ages of 40 and 60, it can be seen in all ages, genders, and ethnicities.

The etiopathogenesis of fibromyalgia involves multiple factors, including dysfunction of the central and autonomic nervous systems, central sensitization, hormonal dysfunction, altered release of neurotransmitters, immune system influence, stress factors, and psychiatric aspects of the disease that come to the fore and are the subject of research.^3^ Because FMS affects the central and peripheral nervous systems as well as the musculoskeletal system, researchers have increasingly focused on balance problems in FMS.^4^ The mechanisms of central sensitization play a crucial role in the development of symptoms and their long-term persistence, affecting various body systems, including postural control.^4^

Postural responses that achieve balance occur through the evaluation of visual, vestibular, and proprioceptive information in the central nervous system.^5^ Static-dynamic posturographic evaluations, balance scales, motor disability tests, and various methods of measuring the patient's movement and walking safety are used in balance evaluation.^6^ It has been reported in current studies that balance problems may occur in FMS.^7,8^ In 1 study, it was revealed that balance problems are one of the leading complaints in FMS and are present in 45% of the patients, while in another study, balance problems were reported as high as 68%.^7,8^ The high rates of balance impairment demonstrate the necessity for thorough evaluation methods in FMS management.

Kinesiophobia, defined as an irrational and debilitating fear of activity or movement due to the possibility of injury or reinjury, is described as excessive and irrational.^9^ Severe exacerbation of pain symptoms following physical activity in FMS is one of the main features of the disease.^10^ It has been reported that for certain patients, the experience of pain results in kinesiophobia (fear of movement), which may cause avoidance of pain-management strategies, and in the long term, kinesiophobia may adversely impact the cluster of symptoms experienced by individuals with chronic pain.^11^ Research on kinesiophobia in fibromyalgia patients has produced different results regarding its prevalence and its impact on disability.^12-15^ The relationship between kinesiophobia and objective balance measures has not been well understood.

The existing literature shows both balance problems and kinesiophobia in FMS but lacks research about their potential relationship. The existing literature contains separate studies of these factors, but no study has evaluated how kinesiophobia affects postural control in this population. The development of rehabilitation strategies for FMS requires knowledge about the relationship between psychological and physical aspects of the condition. Studies dealing with the fear of balance and movement in patients with FMS are very limited in the literature. The present research aims to address this knowledge gap through a comprehensive evaluation of balance disorders from different viewpoints and their connection to kinesiophobia. It was hypothesized that higher levels of kinesiophobia would be associated with greater balance impairments in patients with FMS.

## Methods

In this cross-sectional study, 60 FMS patients and 60 healthy volunteers who visited the Physical Medicine and Rehabilitation clinic of Kahramanmaraş Sütçü İmam University Training and Research Hospital from February 2020 to July 2020, and who fulfilled the ACR (American College of Rheumatology) 2016 FMS Classification criteria, were included.^16^ Sample size was determined using G*Power software version 3.1.9.4 (Heinrich-Heine-Universität Düsseldorf; Düsseldorf, Germany), assuming an effect size of 0.5 (moderate), alpha of 0.05, and power of 0.80 for detecting differences in balance parameters and kinesiophobia levels between groups.^17^ The power calculation used a moderate effect size of 0.5 because this value represents a typical threshold in clinical research when specific effect size data from similar populations are not available or when researchers want to ensure adequate statistical power.^18^ Exclusion criteria included cognitive impairment, pregnancy, vision problems, vestibular-cerebellar disease, neurological diseases that may cause balance-posture disorders (conditions such as hemiplegia, extrapyramidal disorders, ALS (Amyotrophic Lateral Sclerosis), MS (Multiple Sclerosis), and muscular disorders), other conditions that may impair balance-proprioception (peripheral nerve damage in the lower extremities, peripheral neuropathy), patients with orthopedic disorders that prevent standing, individuals with diseases that may cause widespread pain conditions (hypothyroidism, malignancies, diabetes mellitus, rheumatological diseases, etc.), and illiterate individuals who were not considered for the study. The research focused exclusively on female participants because FMS affects women 7 times more than men, and researchers wanted to minimize potential sex-related confounding variables based on previous findings about sex differences in balance control and pain perception.^4^

Age, body mass index, height, weight, marital status, educational status, and the number of falls in the last 6 months were recorded. The Fibromyalgia Impact Questionnaire (FIQ) was given to all individuals participating in the study to measure disease activity, and the Tampa Scale for Kinesiophobia (TSK) was used to evaluate movement-related fear. The Turkish validity and reliability of the FIQ have been established by Sarmer et al^19^ with high internal consistency (Cronbach's alpha = 0.72-0.73) and test-retest reliability. Similarly, the Turkish version of the TSK has been validated by Yilmaz et al^20^ with good test-retest reliability. It has been accepted to classify balance measurements into 2 main groups: static and dynamic; for these measurements, computer-controlled complex devices can be used, as well as simple tests that can be applied in the clinical setting. The functional reach test (FRT), the four-square step test (FSST), and the timed up and go test (TUG)^6^ were used as dynamic balance assessment methods. The TUG has been validated for balance assessment with excellent test-retest reliability (ICC = 0.98). The FSST has established cutoff scores that indicate increased fall risk when performance is >15 seconds, with 85% sensitivity and 88%-100% specificity in various populations. These clinical balance tests have been validated and standardized by previous research.^21,22^ A stabilometry device was used as a static balance evaluation method. Medication use was documented but not controlled in the analysis, as this study aimed to evaluate real-world clinical populations. The study acknowledges that medications used to treat FMS, such as pregabalin, could potentially affect balance performance, which is a study limitation. The study measured physical activity levels but did not include them as covariates in the regression models, which is another limitation that should be considered when interpreting results.

Prior to the study, ethical permission was granted by the Kahramanmaraş Sütçü İmam University Training and Research Hospital Clinical Research Ethics Committee (Decision No. 20, dated January 22, 2020). All participants provided written informed consent before participating in the study.

### Fibromyalgia Impact Questionnaire

It is a scale for evaluating health status and physical function in fibromyalgia patients. Burchardt et al developed a tool to assess the functional status and disease activities of FMS patients.^23^ It contains 10 items in total. The maximum score is 100. High scores are associated with high disease activity.

### Tampa Scale for Kinesiofobia

Although Miller, Kopri, and Todd developed the Tampa Kinesiophobia Scale in 1991, they did not publish it. Vlaeyen et al^11^ published the scale in 1995 after obtaining permission from the researchers who developed the scale. The TSK includes 17 items and was designed to assess the level of fear of movement-reinjury. The assessment tool incorporates injury-reinjury and avoidance-fear components in daily life and work-related physical activities.^20^ A maximum of 63 points and a minimum of 17 points can be obtained in this survey; a score greater than 37 is classified as high-grade kinesiophobia, while a score equal to or lower is classified as low-grade kinesiophobia.

### Timed Up and Go Test

The distance forward 3 meters from a back-supported chair was marked on the floor. Subjects were guided to get up from the chair, move forward to the mark, turn around, come back to the chair, and seat themselves again. The time to complete the course was measured in seconds. Performances longer than 12 seconds were regarded as having a chance of decline.

### Four-Square Step Test

A flat floor was divided into 4 squares using boards. All squares are numbered from 1 to 4. At the beginning of the test, the subjects were asked to stand aligned with the squares 1 and 2. Participants were instructed in successive order (1-2-3-4-4-3-2-1) that both feet should touch each square as quickly as possible, without touching the boards, and on each square's floor. To reinforce the ranking, the participants were given an experiment. The test is repeated if the participant is unable to complete the sequence, loses their balance, and comes into contact with the board. The time starts when the first foot makes contact with the ground in the 1st square and ends with the contact of both feet on the ground of the 1st square after the sequencing. The time obtained determines the score.

### Functional Reach Test

The participant was told to reach as far as possible without losing balance, without taking a step or touching the wall, with his shoulder in 90 degrees flexion, elbow and wrist in neutral position, and fingers in fist position. The difference in distance between the tip of the fist before and after reaching was measured. The measurement was conducted 3 times, and the mean was taken. Values below 25 cm were accepted as medium, and values below 15 degrees were considered to reflect high-grade fall risk.

### Stabilometric Measurements

It is a method that has been used since the early 1990s to evaluate balance function in different diseases. Static posturography has started to be seen as an alternative to classical methods in the evaluation of balance in vestibular diseases, hemiplegia, Parkinson's disease, multiple sclerosis, and ataxia. Bauer et al^21^ proved the reliability of the static posturographic device in a study on elderly individuals. The device has been validated in patients with chronic pain conditions, with intraclass correlation coefficients ranging from 0.76 to 0.91 for the parameters measured.

Stabilometric measurements were performed using a computerized static posturography platform (NeuroCom Balance Master®, Natus Medical Inc., USA), which is widely used in rehabilitation centers across Türkiye. The device specifications included a sampling frequency of 100 Hz, force platform dimensions of 46 × 46 cm, with 4 strain gauge force transducers positioned at each corner. The manufacturer’s guidelines were followed for daily platform calibration before measurements to ensure a zero baseline and verify accuracy with a known 10 kg weight. The measurement protocol followed standardized procedures: participants stood barefoot on the platform with feet positioned shoulder-width apart, arms relaxed at their sides, looking straight ahead at a visual target placed at eye level 2 meters away. Each test condition lasted 30 seconds with 1-minute rest intervals between trials to prevent fatigue.

Eyes open position on stable ground is accepted as a reference in stableometric measurements. The effect of vision on balance is observed with the eyes closed. The somatosensory system is restricted by foam-rubber pads on the moving floor with the eyes open. On the moving floor with the eyes closed, only the vestibular system is active and tested^16^ (Table 1). Throughout this test, the subject remains on a platform, and a series of data is obtained from the pressure sensors according to the change in the center of gravity [Mediolateral velocity of swinging (mm/sec), mediolateral total length of swinging (mm), mediolateral mean length of swinging (mm), anteroposterior velocity of swinging (mm/sec), anteroposterior total length of swinging (mm), anteroposterior mean length of swinging (mm)]. The anteroposterior (AP) mean parameter specifically represents the average displacement of the center of pressure in the anteroposterior direction during the 30-second test period, calculated as the total anteroposterior path length divided by the number of data points collected at a 100 Hz sampling rate.

### Statistical Analysis

Statistical analyses were conducted using SPSS 20.0 program (IBM SPSS Corp.; Armonk, NY, USA). Results were expressed as numbers, percentages, mean ± SD, and median (minimum-maximum). The distribution of data was evaluated with the Shapiro-Wilk test. Comparisons between the 2 groups were made using the Mann–Whitney *U* test because of the distribution of data for continuous variables. The chi-square test was used to compare the categorical variables between the 2 groups. Correlation analyses were performed with the Spearman correlation test according to the distribution of data. The Bonferroni correction method was used to adjust for multiple comparisons when necessary. The study used *P* < .05 as its statistical significance threshold. A post-hoc power analysis was conducted to assess the study's capacity for detecting kinesiophobia and balance parameter correlations.

## Results

A total of 120 female individuals, 60 in the fibromyalgia patient cohort and 60 in the control cohort, were included in the study. The median age of the fibromyalgia cohort was 37 (min. 18 to max. 57) and the control cohort was 35 (min. 21 to max. 61). The demographic profiles of the participants were compared; no significant difference was observed in terms of mean age, mean body mass index, marital status, and educational status (*P* > .05) (Table 2). The medication usage between groups showed substantial variations because FMS patients took pregabalin (30.0%), duloxetine (25.0%), amitriptyline (20.0%), and NSAIDs (Non-Steroidal Anti-Inflammatory Drug) (46.7%), whereas controls used all medications at *P* < .001. Of the 60 FMS patients, 15 (25.0%) used a single medication, 20 (33.3%) used 2 medications concurrently, 15 (25.0%) used 3 or more medications, and 10 (16.7%) used no medication. The physical activity levels between groups showed a significant difference because FMS patients had 58.3% sedentary participants, whereas controls had 30.0% sedentary participants (*P* = .002) (Table 2). While the number of falls in the patient group was 48 in the last 6 months, affecting 30 patients (50% of the FMS group), the control group did not report any falls. The fall distribution showed that 18 patients (30%) experienced 1 fall, 8 patients (13.3%) experienced 2 falls, and 4 patients (6.7%) experienced 3 or more falls during the 6-month period. Fall frequency differed significantly between groups in the last 6 months between the patient and control cohorts (*P* < .001).

A significant difference was identified between the patient and control groups in terms of the FRT, FSST, and TUG assessments applied for the clinical balance assessment of the participants and the TSK score used in the evaluation of kinesiophobia (*P* < .001) (Table 3).

A significant difference was identified between the patient and control groups in the data obtained in all conditions with stabilometric measurements, and the balance data in the patient group was worse (*P* < .05) (Table 4). The Bonferroni correction for multiple comparisons resulted in an adjusted α = 0.002, which maintained significance for most posturographic parameters, especially in the “unsteady floor, opened eyes” and “unsteady floor, closed eyes” conditions, where 11 of 12 parameters remained significant (Table 4).

In the stabilometric evaluation of balance on a stable surface with eyes closed, a positive statistical correlation was observed between stationary floor, closed eyes (SCE) mediolateral meanlength of swinging (MEAN), SCE AP VEL, SCE AP total length of swinging, SCE AP MEAN data and FIQ scores (rho: 0.275; 0.283; 0.279; 0.406) (*P* < .05 for all). However, after Bonferroni correction, only the correlation between SCE AP MEAN and FIQ remained significant (rho = 0.406, adjusted *P* = .024) (Table 5). There was no statistically significant correlation between the results of the stabilometric measurements of balance under “stationary floor, opened eyes,” “unsteady floor, opened eyes,” “unsteady floor, closed eyes” conditions, and FIQ scores (*P* > .05). A statistically significant correlation was observed between TSK scores and FIQ scores (*r*: 0.507, *P* < .001). A statistically significant correlation was observed between the number of falls in the last 6 months and FIQ scores (*r*: 0.256, *P* < .001). Both correlations remained significant after Bonferroni correction (adjusted *P* < .024) (Table 5).

There was no statistically significant relationship between the TSK scores used to measure the kinesiophobia levels of the patient and control groups, and the FRT, FSST, TUG scores used for the clinical balance measurement, and the stabilometric measurement data (*P* > .05). The post-hoc power analysis showed that this study had 84% power to detect medium effect sizes (*r* ≥ 0.3) but only 54% power to detect small effect sizes (*r* ≥ 0.2). The observed correlations between TSK and balance measures ranged from *r* = 0.08 to *r* = 0.15, suggesting that detecting these small effects would require approximately 350 participants to achieve 80% power.

The multivariate regression analysis (Table 6) showed that age (β=0.354, *P* < .001) and FIQ score (β = 0.246, *P* = .012) were significant predictors of TUG performance (R² = 0.287). For FSST, age (β = 0.298, *P* = .002) and FIQ score (β = 0.213, *P* = .045) were also significant predictors (R² = 0.259). TSK scores did not significantly predict any balance parameter in the regression models. Logistic regression for fall risk showed that FIQ score (OR = 1.138, 95% confidence interval [CI]: 1.024-1.265, *P* = .015) and TUG performance (OR = 1.254, 95% CI: 1.001-1.571, *P* = .049) were significant predictors of falls in FMS patients (Table 6). There was a negative correlation between age and FRT and a positive correlation with TUG (rho:−0.302; 0.354)(*P* < .05), but there was no significant correlation with FSST (*P* > .05).

## Discussion

This cross-sectional study demonstrated that female patients with fibromyalgia syndrome had significantly impaired balance and higher kinesiophobia compared to healthy controls. Significant differences were found in clinical balance measures, posturographic parameters, and fall frequency between groups, but no significant correlation between kinesiophobia and balance parameters, despite both being affected in FMS. This unexpected finding suggests that these phenomena operate through independent mechanisms in this patient population, with important implications for clinical practice and rehabilitation approaches.

The research findings support existing knowledge about balance problems in FMS patients while providing new insights through comprehensive assessment methods. The study evaluated balance dysfunction in this population through clinical tests, posturographic evaluation, and fall history assessment.

The current literature has shown that there is a higher risk of falling in FMS compared to healthy individuals. Jones et al^24^ and Meireles et al^25^ reported the average number of falls in 6 months as 1.15 and 1.65, respectively. Similarly, in the current study, the average number of falls in the last 6 months in FMS patients was determined to be 0.8.

Clinical tests such as the Berg Balance Test (BBT), Activities-specific Balance Confidence Test (ABC), FRT, TUG, and FSST are still popular in the evaluation of balance.^22^ Santo et al^26^ reported that the results of BBT and ABC tests were worse in the FMS group. Costa et al^27^ reported that the results of the TUG and BBT of the FMS cohort were worse, and the balance was more disturbed. In the current study, there was a significant increase in TUG and FSST durations in the FMS cohort compared to the control cohort, while there was a significant decrease in FRT distances. The observed differences between groups showed large to medium effect sizes (Cohen's d: 0.68-0.89), which indicated both statistical significance and clinical importance. The 2.55 seconds difference in TUG performance between groups surpassed the established minimal clinically important difference of 2.2 seconds for older adults, but FMS-specific thresholds need further determination.

The clinical balance test cutoff values in this study (such as 12 seconds for TUG and 25/15 cm for FRT) were derived from research in other populations, primarily older adults. While these measures have been used in previous FMS studies, there are currently no FMS-specific validated cutoff scores. This represents a limitation in the field, as these thresholds may not have the same clinical significance in younger FMS patients as they do in geriatric populations. Future research should work to establish FMS-specific normative values for these widely used clinical tests.^28^

Computed posturography measurements are an increasingly important method in the evaluation of balance.^21^ Jones et al^4^ in their study on FMS and control groups using a dynamic posturography device, reported significant deterioration in the Sensory Organization Test (SOT) balance scores in the FMS group (in all conditions other than “stationary floor, opened eyes” [SOE]). Collado-Mateo et al^29^ reported that SOT scores were significantly impaired in the FMS group. In this study, the data obtained with the posturography device under all conditions are significant in favor of balance disorders in the FMS group. The obtained data are consistent with the literature.

One of the factors leading to pain and disability in fibromyalgia is seen as kinesiophobia. In various studies evaluating kinesiophobia in FMS patients, kinesiophobia was reported as 72.9% by Russek et al,^30^ 38.6% by Turk et al,^31^ and 75.1% by Koçyiğit et al.^32^ In the current study, 81.7% of the patients had high kinesiophobia, with a median TSK value of 44 (min. 29 to max. 60). The results of this study show that the majority of FMS patients have high levels of kinesiophobia.

The relationship between falls and disease activity in FMS warrants careful examination. This research confirms the findings of Collado-Mateo et al^28^ and supports Russek and colleagues’^29^ theoretical framework about symptom severity effects on postural control through the discovery of a significant correlation between FIQ scores and fall frequency (*r* = 0.256, *P* < .001). The complex pathophysiology of FMS leads to sensorimotor integration impairment through multiple mechanisms that include central sensitization effects on proprioception and pain-related attention demands, and muscle function impairments from fatigue and medication side effects. The relationship between FMS and falls shows stronger links to central nervous system changes and total symptom severity compared to osteoarthritis, which primarily experiences falls because of mechanical factors. The regression analysis confirms that FIQ and TUG serve as important predictors of falls because they demonstrate the complex relationship between disease activity, functional mobility, and fall risk in this population.^33^

Russek et al^30^ determined a meaningful negative correlation between the scores of the SOT assessment in the SCE condition and the FIQ score in the FMS group. Collado-Mateo et al^29^ determined a significant positive correlation between the FIQ level and the number of falls in the last 6 months. In the current study, a statistically significant but weak positive correlation was observed between anteroposterior sway data and FIQ score in posturography measurement under the SCE condition. Additionally, a weak yet statistically significant correlation was found between the FIQ score and the number of falls in the last 6 months. These findings suggest that an increase in FMS disease activity may contribute to balance disorders.

There are some studies evaluating the relationship between kinesiophobia and disease activity in fibromyalgia. Previous studies reported a statistically significant relationship between TSK level and FIQ level.^30,32^ In the current study, similar to previous studies, a statistically significant relationship was found between the TSK score and FIQ score.

In the current study, no significant relationship was observed between kinesiophobia and balance. There are a few studies in the literature examining the relationship between kinesiophobia and balance. One of these studies demonstrated the existence of this relationship in patients with patellofemoral pain syndrome (PFPS).^34^ This difference can be explained by the fact that fibromyalgia is related to widespread pain and central mechanisms, while PFPS is related to local biomechanical disorders. Asiri et al^15^ conducted a study that evaluated the relationship between kinesiophobia and balance. The study concluded that kinesiophobia and balance were related, and that balance deteriorated as kinesiophobia increased.^15^ The differences between studies may be due to methodological approaches and sample characteristics. In Asiri et al’s^15^ studies, only static balance assessment methods were used, while in the current study, both static and dynamic balance assessment methods were used. The mean age of the FMS group was 51.52 ± 7.73 in their study and 36.0 ± 9.40 in the current study. Age may have an effect on both balance and kinesiophobia.

The study results showed no connection between kinesiophobia and balance parameters despite both conditions being present in FMS patients. The central nervous system complexity in FMS could explain this finding because central sensitization impacts multiple systems through different pathways. The cognitive-affective pathways of fear-avoidant behaviors (kinesiophobia) differ from the sensorimotor integration deficits and altered proprioceptive processing that affect balance impairments. The fear-avoidance model, which explains chronicity in many pain conditions, shows a different expression in FMS because of its extensive neurophysiological impacts.^32^

The application of Bonferroni correction in the correlation analyses, while necessary to control type 1 error, may have obscured potentially meaningful small correlations between kinesiophobia and balance measures. This conservative approach represents a trade-off between statistical rigor and sensitivity to detect subtle relationships. The observed correlations (*r* = 0.08-0.15) between TSK and balance parameters, though non-significant, might reflect genuine but weak associations that require larger sample sizes to detect reliably.

The study failed to evaluate psychological elements such as depression and anxiety, which commonly affect FMS patients and affect kinesiophobia and balance performance separately. The relationship between kinesiophobia and balance could be affected by unmeasured variables, which might also influence their impact on functional outcomes. Future research needs to include complete psychological evaluations to understand these intricate relationships better.^35^

The post-hoc power analysis showed that detecting effect sizes of *r* ≥ 0.2 would need approximately 350 participants to reach 80% power. Future research on the relationship between kinesiophobia and balance in FMS should use substantially larger sample sizes to detect potentially clinically meaningful but statistically small associations. 

The treatment outcomes of FMS require special attention to chronic pain acceptance.^36^  Research indicates that psychological acceptance of chronic pain strongly affects the treatment adherence and exercise participation of FMS patients.^37^ Research indicates that patients who accept their pain better show improved physical therapy participation and functional results.^36,37^ These findings could be influenced by individual differences in pain acceptance levels because the lack of correlation between kinesiophobia and balance might be moderated by these differences. Patients who have learned to accept their pain condition will maintain better balance performance even with high kinesiophobia scores because they continue participating in physical activities instead of avoiding them.^36,37^ Future rehabilitation programs should combine acceptance-based psychological interventions with balance training to achieve the best possible treatment results.

The study failed to account for medications used by FMS patients, which creates a major research limitation. The medications prescribed to FMS patients, which include pregabalin (30.0%), duloxetine (25.0%), amitriptyline (20.0%), and NSAIDs (46.7%), could influence both balance performance and kinesiophobia levels. The heterogeneous medication regimens and polypharmacy patterns (58.3% using multiple medications) in the study sample might have masked actual relationships between the studied variables. The regression analyses failed to account for the substantial difference in physical activity levels between groups because 58.3% of FMS participants were sedentary, while only 30.0% of controls were sedentary, thus creating another potential confounding factor.

The self-reported falls during a 6-month period might contain recall bias, particularly problematic given that FMS patients commonly experience cognitive problems known as "fibro fog." The cognitive impairment might interfere with their ability to correctly remember and document their falls. Research indicates that fibro fog affects between 80% and 100% of FMS patients, and it severely impairs their memory function. The fall frequency data suffers from limited accuracy because objective fall tracking methods, such as daily logs, diaries, and wearable devices, were not used.

The measurement of vitamin D levels across different months could potentially introduce seasonal variations as a confounding factor, though these levels were not included in the primary analyses.

The post-hoc power analysis showed that the sample size was sufficient for detecting medium effect sizes (*r* ≥ 0.3) with 84% power, but it only provided 54% power for small effect sizes (*r* ≥ 0.2). The observed kinesiophobia and balance measure correlations were small (*r* = 0.08-0.15), which indicates that any potential relationship might be too subtle for the sample size to detect reliably. A study with increased participant numbers would be required to determine if small yet important connections exist between these variables.

This study found significant balance deficits in FMS patients compared to healthy controls as measured by clinical balance tests, posturography measurements, and an increased number of falls. Although higher levels of kinesiophobia were seen in FMS patients, no significant association was found between kinesiophobia and balance deficits. The research indicates that FMS patients experience 2 separate symptoms that require different treatment methods.

The study results indicate that FMS rehabilitation programs should include balance training as a treatment method to address the specific deficits found in this research. The high kinesiophobia rate (81.7%) among the FMS patients could affect treatment compliance, so movement-related fears need simultaneous attention with balance training for better results. Future intervention studies are needed to determine the efficacy of such approaches. Combining balance exercises with traditional aerobic and stretching programs may contribute to improving postural stability. It should not be forgotten that kinesiophobia is a factor that should be considered in the treatment of FMS disorders. The research findings demonstrate that complete evaluation methods combined with treatment plans that handle physical and psychological elements of this complex condition are essential.

## Figures and Tables

**Table 1.** Stabilometer Measurement Conditions

Position	Floor	Eyes	Aim
SOE	On the platform	Opened	Evaluation of static posture
SCE	On the platform	Closed	Elimination of the visual system
UOE	On the sponge on the platform	Opened	Elimination of the somatosensory system
UCE	On the sponge on the platform	Closed	Elimination of somatosensory and visual systems

SCE, stationary floor, closed eyes; SOE, stationary floor, opened eyes; UCE, unsteady floor, closed eyes; UOE, unsteady floor, opened eyes.

**Table 2.** Comparison of Socio-Demographic Data of Fibromyalgia Syndrome and Control Groups

	FMS (n = 60)	Control (n = 60)	**P**
Age*(median) (minimum-maximum)	37 (18-57)	35 (21-61)	.472
BMI (kg/m²) (median) (minimum-maximum)	26.55 (19-36)	26 (19-37)	.567
Marital status (n) Married Single	53 7	43 17	.572
Educational Status (n) Primary education High school University Master/PhD	37 14 7 2	33 16 7 4	.794
Occupation (n) Employee Unemployed	21 39	24 36	.572

BMI, body mass index; FMS, fibromyalgia syndrome; n, number.

**Table 3.** Clinical Balance Assessments and Kinesiophobia Scores

Parameters	FMS (n = 60)	Control (n = 60)	*P*
TSK score	44 (29-60)	34 (17-45)	<.001*
TUG (sec)	10.80 (8-16)	8.25 (6-13)	<.001*
FSST (sec)	12.75 (9-18)	9.60 (8-17)	<.001*
FRT (cm)	31.50 (20-48)	37 (20-50)	<.001*

Data expressed as median (minimum-maximum); statistical analysis: Mann–Whitney *U* test.

FMS, fibromyalgia syndrome; FRT, functional reach test; FSST, four-square step test; TSK, Tampa Scale for Kinesiophobia; TUG, timed up and go test.

*Statistically significant at *P* < .05.

**Table 4.** Comparison of Posturography Data of Fibromyalgia Syndrome and Control Groups

	FMS (n = 60)	Control (n = 60)	**P**
SOE ML VEL	10.95 (2-46)	9.21 (2-12)	.001*
SOE ML TOT	312.50 (68-1323)	266 (58-344)	<.001*
SOE ML MEAN	1.60 (1-5)	1.45 (0-2)	.002*
SOE AP VEL	11.50 (3-29)	10.08 (2-15)	.026*
SOE AP TOT	332 (77-831)	289.50 (65-421)	.012*
SOE AP MEAN	1.29 (1-3)	1.21 (1-2)	.001*
SCE ML VEL	11.25 (5-50)	9.74 (4-17)	.011*
SCE ML TOT	317 (14-1348)	279 (119-476)	.046*
SCE ML MEAN	1.75 (1-5)	1.59 (1-5)	.002*
SCE AP VEL	14.32 (6-44)	11.96 (2-17)	<.001*
SCE AP TOT	409.50 (182-1270)	342.50 (77-497)	<.001*
SCE AP MEAN	1.72 (1-7)	1.47 (1-3)	<.001*
UOE ML VEL	13.07 (5-45)	10.47 (3-17)	<.001*
UOE ML TOT	380.50 (208-1294)	301.50 (96-497)	<.001*
UOE ML MEAN	2.72 (2-10)	1.96 (1-4)	<.001*
UOE AP VEL	14.18 (2-34)	11.64 (2-24)	<.001*
UOE AP TOT	406.50 (254-958)	337.50 (88-695)	<.001*
UOE APMEAN	1.68 (1-4)	1.51 (1-6)	<.001*
UCE ML VEL	20.60 (10-66)	16.22 (4-36)	<.001*
HCE ML TOT	590.50 (180-1875)	471 (47-1025)	<.001*
HCE ML MEAN	5.73 (3-15)	4.39 (1-11)	<.001*
HCE AP VEL	28.99 (14-67)	24.60 (5-44)	.005*
HCE AP TOT	788 (421-1917)	682 (100-1244)	.001*
HCE AP MEAN	5.42 (2-19)	4.39 (1-13)	.007*

Data are expressed as median (minimum-maximum).

AP, anteroposterior; FMS, fibromyalgia syndrome; MEAN, meanlength of swinging (mm); ML, mediolateral; SCE, stationary floor, closed eyes; SOE, stationary floor, opened eyes; TOT, total length of swinging (mm); UCE, unsteady floor, closed eyes; UOE, unsteady floor, opened eyes; VEL, velocity of swinging (mm/sec).

*P < .05.

**Table 5.** Correlation Analysis of Fibromyalgia Impact Questionnaire Scores and Posturography Measurements

		SOE ML VEL	SOE ML TOT	SOE ML MEAN	SOE AP VEL	SOE AP TOT	SOE AP MEAN
FIQ	Rho	−0.087	−0.085	0.147	0.013	0.061	0.002
	*P*	.507	.516	.264	.922	.646	.989
		SCE ML VEL	SCE ML TOT	SCE ML MEAN	SCE AP VEL	SCE AP TOT	SCE AP MEAN
FIQ	Rho	0.013	−0.048	0.275	0.283	0.279	0.406
	*P*	.919	.717	.034*	.028*	.031*	.001*
		UOE ML VEL	UOE ML TOT	UOE ML MEAN	UOE AP VEL	UOE AP TOT	UOE AP MEAN
FIQ	Rho	0.004	−0.043	0.088	0.147	0.125	0.227
	*P*	.974	.742	.504	.263	.341	.082
		UCE ML VEL	UCE ML TOT	UCE ML MEAN	UCE AP VEL	UCE AP TOT	UCE AP MEAN
FIQ	Rho	0.100	0.043	0.094	0.102	0.134	0.164
	*P*	.449	.745	.473	.437	.308	.212

Rho, correlation analysis coefficient.

AP, anteroposterior; FIQ, Fibromyalgia Impact Questionnaire; MEAN, mean length of swinging (mm); ML, mediolateral; SCE, stationary floor, closed eyes; SOE, stationary floor, opened eyes; TOT, total length of swinging (mm); UCE, unsteady floor, closed eyes; UOE, unsteady floor, opened eyes; VEL, velocity of swinging (mm/sec).

*Statistically significant at the *P* < .05 level.

**Table 6.** Multivariate Regression Analysis of Factors Associated with Balance Parameters and Fall Risk in Fibromyalgia Patients

Variables	Model 1: TUG	Model 2: FSST	Model 3: FRT	Model 4: Falls
	β (95% CI)	β (95% CI)	β (95% CI)	OR (95% CI)
Age (years)	0.354*** (0.193-0.515)	0.298** (0.116-0.480)	-0.302 (-0.614-0.010)	1.042 (0.986-1.101)
FIQ score	0.246* (0.058-0.434)	0.213* (0.005-0.421)	-0.268 (-0.552-0.016)	1.138* (1.024-1.265)
TSK score	0.108 (-0.094-0.310)	0.095 (-0.141-0.331)	-0.042 (-0.378-0.294)	1.025 (0.953-1.102)
BMI (kg/m²)	0.089 (-0.132-0.310)	0.126 (-0.124-0.376)	-0.195 (-0.543-0.153)	–
Number of falls	0.182 (-0.012-0.376)	0.205 (-0.011-0.421)	-0.312* (-0.608--0.016)	–
TUG (sec)	–	–	–	1.254* (1.001-1.571)
FSST (sec)	–	–	–	1.186 (0.896-1.570)
FRT (cm)	–	–	–	0.942 (0.857-1.035)
				
Model statistics				
R²	0.287	0.259	0.241	0.342^a^
Adjusted R²	0.254	0.225	0.206	–
F-statistic	8.342***	7.124***	6.542***	–
*P*	<.001	<.001	<.001	–

Models 1-3: Linear regression; Model 4: Binary logistic regression; Dashes indicate variables not included; Post-hoc power analysis: With n = 60/group and α = 0.05, study had 84% power for medium effects (*r* ≥ 0.3) but 54% for small effects (*r* ≥ 0.2).

BMI, body mass index; CI, confidence interval; FIQ, Fibromyalgia Impact Questionnaire; FRT, functional reach test; FSST, four-square stepping test; OR: odds ratio; TSK, Tampa Scale for Kinesiophobia; TUG, timed up and go test; β, unstandardized coefficient.

^a^Nagelkerke R^2^; Hosmer–Lemeshow test: χ²(8) = 9.124, *P* = .332.

*P < .05.

**P < .01.

***P < .001.

## Data Availability

The data that support the findings of this study are available on request from the corresponding author.
